# Demography and the Age of Rare Variants

**DOI:** 10.1371/journal.pgen.1004528

**Published:** 2014-08-07

**Authors:** Iain Mathieson, Gil McVean

**Affiliations:** Wellcome Trust Centre for Human Genetics, University of Oxford, Oxford, United Kingdom; University of Chicago, United States of America

## Abstract

Large whole-genome sequencing projects have provided access to much rare variation in human populations, which is highly informative about population structure and recent demography. Here, we show how the age of rare variants can be estimated from patterns of haplotype sharing and how these ages can be related to historical relationships between populations. We investigate the distribution of the age of variants occurring exactly twice (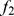 variants) in a worldwide sample sequenced by the 1000 Genomes Project, revealing enormous variation across populations. The median age of haplotypes carrying 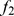 variants is 50 to 160 generations across populations within Europe or Asia, and 170 to 320 generations within Africa. Haplotypes shared between continents are much older with median ages for haplotypes shared between Europe and Asia ranging from 320 to 670 generations. The distribution of the ages of 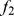 haplotypes is informative about their demography, revealing recent bottlenecks, ancient splits, and more modern connections between populations. We see the effect of selection in the observation that functional variants are significantly younger than nonfunctional variants of the same frequency. This approach is relatively insensitive to mutation rate and complements other nonparametric methods for demographic inference.

## Author Summary

In this paper we describe a method for estimating the age of rare genetic variants. These ages are highly informative about the extent and dates of connections between populations. Variants in closely related populations generally arose more recently than variants of the same frequency in more diverged populations. Therefore, comparing the ages of variants shared across different populations allows us to infer the dates of demographic events like population splits and bottlenecks. We also see that rare functional variants shared within populations tend to have more recent origins than nonfunctional variants, which is consistent with the effects of natural selection.

## Introduction

The recent availability of large numbers of fully sequenced human genomes has allowed, for the first time, detailed investigation of rare genetic variants. These are highly differentiated between populations [1], [2], may make an important contribution to genetic susceptibility to disease [3]–[7], and provide information about both demographic history, and fine-scale population structure [8], [9]. While patterns of rare variant sharing are informative in themselves, knowing the age of the variants allows us to observe changes in structure over time, and thus to infer the dates of demographic events.

Rare variants are typically more recent than common variants and in fact, the age of a variant can be estimated directly from its frequency [10]–[12]. However there are two problems with this approach. First, using only the frequency information means that we cannot distinguish differences between the ages of variants which are at the same frequency which, as we demonstrate here, can be both large and important. Second, in order to use this approach, we have to know the demographic history of the populations involved. In this article, we describe an alternative approach which uses the fact that the lengths of shared haplotypes around variants are informative about their ages [13]–[15].

Specifically, we estimate the time to the most recent common ancestor (TMRCA) for 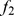 haplotypes, which are regions where two chromosomes are each other's closest relative in a sample. More precisely, 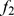 haplotypes are genomic regions where two chromosomes are each other's unique closest relative within at least some of the region and where their TMRCA is constant. To find these regions, we look for variants which occur exactly twice in the sample (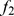 variants, or doubletons). We then use the length of, and number of mutations on, these haplotype to infer their ages, and therefore a lower bound for the age of the variants they carry. Every 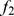 variant identifies an 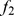 haplotype, but we do not detect all 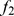 haplotypes because not all of them carry mutations. This approach is highly scalable and finds shared haplotypes directly from genotype data, which avoids the need for statistical phasing. We apply this method to the 1000 Genomes phase 1 dataset [16], to quantify the distribution of the ages of variants shared within and between populations, and between variants in different functional classes. We demonstrate dramatic differences between the ages of variants shared across different populations, and observe the effects of both demography and selective constraint.

## Results

**Figure 1:**
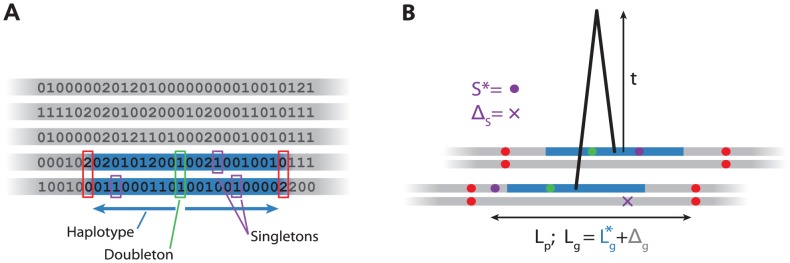
Algorithm and model for haplotypes. **A**: Algorithm for detecting 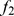 haplotypes. For each 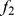 variant in the sample (green), we scan left and right until we find inconsistent homozygote genotypes (red), record the physical and genetic length of this region (blue), and the number of singletons (purple). **B**: Model for haplotype age 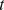. Consider the 4 chromosomes (grey) of the two individuals sharing an 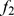 haplotype (blue). We model the total genetic length of the inferred haplotype, 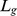, as the sum of the true genetic length 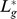 and an error 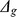. Similarly, we model the number of singletons 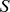 as the sum of the number on the shared chromosome (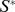) and the number on the unshared chromosomes, 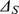. We ignore the fact that we overestimate 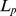 and therefore that some of the singletons might lie in the unshared part of the chromosome.

We first give a brief outline of our approach (Figures 1, S1, **Methods**). Given a sample of individual genotypes, we find all 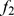 variants. That is, variants which have exactly two copies (in different individuals) in the sample. This tells us that, in the absence of repeat mutations and assuming that the 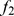 variant is derived, those individuals must share an 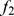 haplotype at that position. We then scan left and right along the genome, until we reach a point where the two individuals have inconsistent homozygote genotypes (0 and 2, Figure 1A), which gives us an (over-) estimate of the distance to the first recombination breaking the haplotype.

Using both the genetic and physical lengths of the region, and the number of singletons, we compute an approximate likelihood for the age of the haplotype (Figure 1B). We use the data to estimate error terms to take into account the fact that the algorithm described above does not find the shared haplotypes precisely. Then, for each haplotype, we find the maximum likelihood estimate (MLE) of the age of each haplotype. We investigate the distribution of these MLEs for different classes of 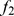 variants, for example those shared within or between specific populations.

### Simulation results

**Figure 2:**
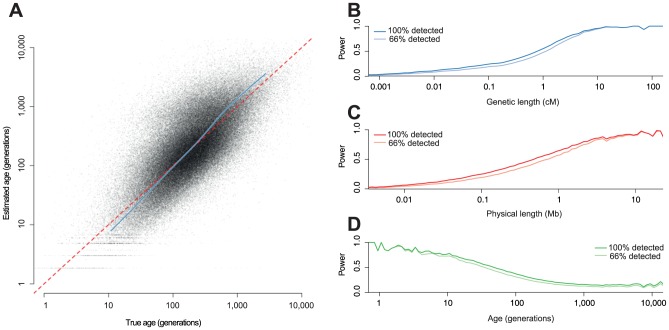
Estimating 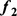 age from simulated data. We simulated whole genomes for 100 individuals (200 chromosomes), with 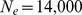, 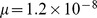 and HapMap 2 recombination rates. **A**: Estimated age against true age. The grey dots are the MLEs for each detected haplotype. The blue line is a quantile-quantile (qq) plot for the MLEs (from the 1*^st^* to 99*^th^* percentile). **B–D** Power to detect 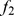 haplotypes as a function of **B**: genetic length, **C**: physical length and **D**: haplotype age; in each case the darker line represents the power to detect 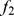 haplotype with 100% power to detect 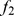 variants, and the lighter line the power with 66% power.

To test our approach, we ran whole genome simulations for a sample of 100 diploid individuals with MaCS [17], using the combined HapMap 2 recombination maps [18], and a mutation rate (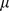) of 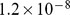 per-base per-generation, assuming a constant effective population size (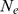) of 14,000; chosen to reflect parameters relevant to human genetic variation. We investigated both our power to detect the 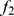 haplotypes and how accurately we could estimate the distribution of 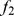 ages (Figure 2). We detected around 26% of all 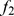 haplotypes. Unsurprisingly, we have more power to detect very long haplotypes, but we detected many small haplotypes as well: 19% of our total had true genetic length less than than 0.1 cM. Having imperfect power to detect 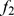 variants does not have a large effect on our power to detect 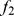 haplotypes since most detected haplotypes carry more than one 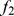 variant. We have higher power for more recent haplotypes because they are longer but, at least for a population of constant size, this effect is cancelled to some extent for older haplotypes because the branches above them tend to be longer and therefore more likely to carry mutations.

There is high uncertainly in the age of any individual haplotype (Figure 2A). However, we can compute well-calibrated confidence intervals (Figure S2). In this example, the median MLE of the age of the detected haplotypes is 179 generations and the true median is 192 generations. The median width of the 95% confidence interval is 730 generations. Information about the ages comes mainly from the genetic length, and the principal advantage of the singleton information is for very old haplotypes where the length-based estimator is otherwise biased (Figure S3).

In addition, we ran simulations to check that the model was robust to more complicated demographic scenarios including splits, bottlenecks and expansions, as well as mis-specification of 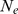 (Figure S4). We also investigated the effect that these scenarios had on the distribution of the ages of the 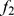 haplotypes, demonstrating that we could detect the signatures of demographic events. For example, population bottlenecks lead to a high density of 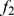 haplotypes during the bottleneck and, following a population split haplotypes shared between populations have median age roughly equal to the split time (Figure S5).

### 1000 Genomes data

We applied our estimator to the phase 1 data release of the 1000 Genomes Project [16], which consists of whole-genome variant calls for 1,092 individuals drawn from 14 populations (Table 1). We used two of the 1000 Genomes callsets; one made from sequence data, and one made using a dense genotyping array. Restricting our analysis to the autosomes, we extracted 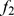 variants from the sequence data callset, and then detected haplotype lengths around them (that is, the distances to incompatible homozygotes), using only the array data, to minimise the effect of genotyping errors. We then counted 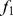 variants on these haplotypes from the sequence data callset. From 4,066,530 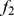 variants we detected 1,893,391 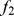 haplotypes, with median genetic and physical lengths of 0.7 cM and 600 kb respectively. The median number of singletons spanned by each 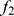 haplotype was 3. Of the 1.9 million 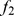 haplotypes, 0.7 million were shared within populations and 1.5 million were shared within continents. Sharing of 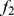 variants largely reflects expected patterns of relatedness on a population level, and also reveals substructure in some populations, notably GBR (Figure S6).

**Table 1:** Short descriptions of the 1000 Genomes populations.

Abbreviation	Sample size	Description
ASW	61	African Ancestry in SW USA
LWK	97	Luhya in Webuye, Kenya
YRI	88	Yoruba in Ibadan, Nigera
CLM	60	Colombians in Medellín, Colombia
MXL	66	Mexican Ancestry in Los Angeles, CA, USA
PUR	55	Puerto Ricans in Puerto Rico
CHB	97	Han Chinese in Beijing, China
CHS	100	Han Chinese South
JPT	89	Japanese in Tokyo, Japan
CEU	85	Utah residents with ancestry from northern and western Europe
FIN	93	Finnish in Finland
GBR	89	British from England and Scotland
IBS	14	Iberian Populations in Spain
TSI	98	Toscani in Italy

**Figure 3:**
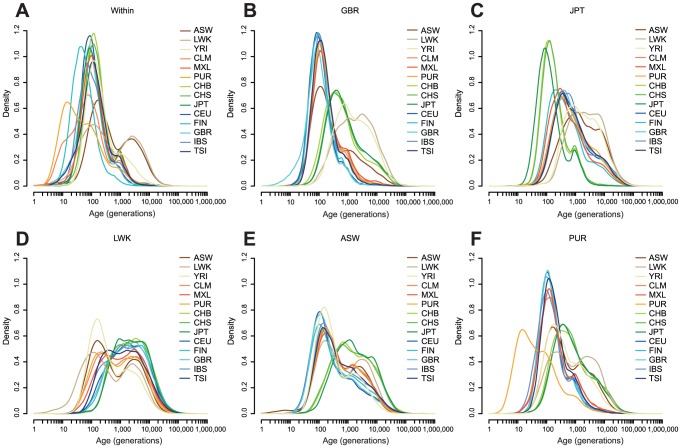
The estimated age distribution of 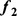 haplotypes. **A**: The distribution of the MLE of the ages of haplotypes shared within each population. **B–F**: The distribution of the MLE of the ages of haplotypes shared between one population and all other populations, shown for each of GBR, JPT, LWK, ASW, and PUR. Populations are described in Table 1. Density estimates are computed in 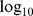 space, using the base R *density* function with a Gaussian kernel.

We used the combined recombination rate map from HapMap 2 to determine genetic lengths, and assumed a mutation rate of 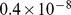 per-base per-generation (reflecting a true mutation rate of 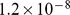 multiplied by a power of 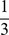 to detect singletons [16], [19]). We then computed MLEs for the ages of all the 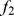 haplotypes shared between every pair of populations (Figures 3, S7, Tables S1–S5). By considering the distribution of the distance between inconsistent homozygous SNPs, we estimated that on most chromosomes the median overestimate in haplotype length due to the sparsity of informative SNPs was 0.1–0.15 cM (but more on chromosomes 1, 9 and 15). We also estimated that 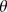 (estimated from singletons) was around 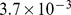 and 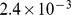 per-base for African and Non-African populations respectively (Table S6).

For haplotypes shared within populations (Figure 3A), the MLEs of haplotypes within most European and Asian populations are clustered around 100 generations ago. For example, the median age of GBR-GBR haplotypes is 90 generations. PUR and, to a lesser extent, CLM have many very recent haplotypes (peaking around 11 generations ago), consistent with a historical bottleneck in these populations 300–350 years ago. FIN haplotypes peak around 14 generations (400–450 years) ago. African populations have many recent haplotypes but also a much longer tail than the other populations, with ancestry apparently extending back for thousands of generations. For example the median age of LWK-LWK haplotypes is 320 generations, but the 95% quantile is 8,500 generations.

Between-population sharing is largely consistent with the historical relationships among populations (Figure 3B–D). Within continents, sharing within Asia or Europe has a median of 50–160 generations, depending on the populations, and sharing within Africa 170–340 generations. Sharing between continents is much older, with median Asian-European sharing 320–670 generations old, and Asian-African sharing rather older, with a median around 2,300 generations ago for LWK and 1,700 generations ago for YRI. The age of European-African sharing varies between populations, from 1,000 to 2,000 generations ago, but is more recent than Asian-African sharing, perhaps suggesting greater subsequent migration between these continents. We discuss these figures in the context of split times and migrations in the **Discussion**.

Admixed populations have age distributions that are combinations of the distributions of the admixing populations (Figure 3E–F). Even in these populations we can see signs of more subtle history. For example, GBR-CLM haplotypes have an age distribution which looks more like GBR-TSI or GBR-IBS than GBR-CEU, presumably representing the fact that the major contribution to European admixture in the Americas is from southern Europe (Figure S8).

**Figure 4:**
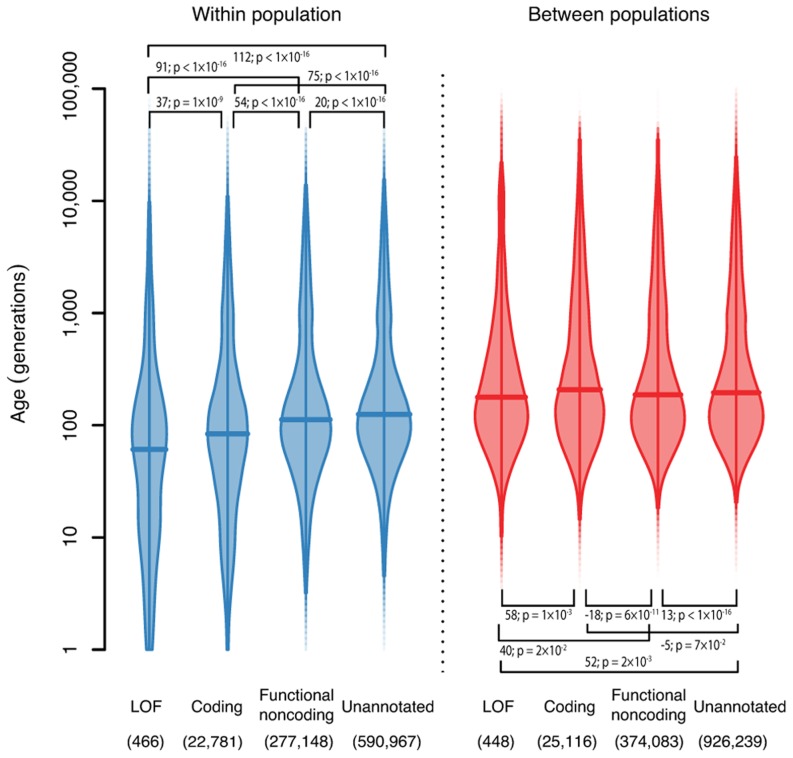
The ages of haplotypes around 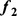 variants with different functional annotations. Density is indicated by the width of the shape, and horizontal bars show the median. We show separately the densities for 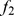 variants shared within a population (left, blue), and 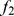 variants shared between populations (right, red). Numbers in brackets show the number of variants in each class. Bars show the pairwise differences in means, and 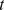 test p-values for a difference in log means between groups.

We also looked at the distribution of the ages of 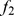 variants broken down by functional annotation (Figure 4, **Methods**). We found that for variants shared within a single population, loss-of-function (LOF) variants are younger than coding variants, which are younger than functional noncoding variants, and all annotated variants are younger than unannotated variants. The median ages of these variants are 58, 83, 112 and 125 generations for LOF, coding, functional noncoding and unannotated variants respectively. This is presumably because purifying selection against damaging mutations means that functional variants are less likely to become old (though positive selection for beneficial mutations would have the same effect). This effect has previously been both predicted and observed [20],[21]. However, it is not strictly true for variants shared between different populations and, in fact, the effect is partially reversed (median ages are 176, 205, 186 and 195 generations for LOF, coding, functional noncoding and unannotated variants). One possible explanation is that functional variants surviving long enough to be shared between populations are selectively neutral or recessive and thus unaffected by selection at low frequency. This suggests that studies looking for disease-causing rare variants should concentrate on variants private to a single population, since variants shared across populations are unlikely to have large phenotypic effects.

### Robustness

This analysis requires us to estimate several parameters, and in this section, we investigate how robust it is to varying them.

The parameter 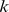 is related to the probability of discovering 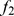 haplotypes. We know that 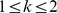. 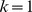 implies that the probability that we discover a haplotype is independent of its length while 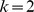 implies that this probability increases linearly with length. We chose 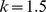 based on simulations, but it may be the case that this is not optimal for real data. To test how much of an impact this might have, we re-ran the analysis of the 1000 Genomes data using 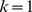 and 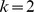. Larger values of 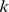 increase our age estimates. For example, the median CEU-CHB age is 403, 481 and 560 generations using 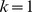, 1.5 and 2 respectively. Overall, setting 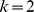 increases the median age estimates by between 6 and 30%, depending on population, with more recent ages more sensitive to 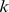.

The parameters 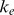 and 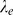 are the shape and rate of the (gamma) distribution of the overestimate of haplotype lengths (**Methods**). We estimated these parameters separately from the array data for each chromosome (Table S6). We noticed that chromosomes 1, 5 and 9 had estimated parameters that implied a greater overestimate (larger 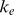, smaller 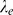), presumably due to the density of markers on the array for those chromosomes. In addition, these chromosomes had older estimated haplotype ages, for example we estimated that the median age of 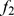 haplotypes on chromosome 1 was 16% higher than the median age of haplotypes on chromosome 2, suggesting that our error model is not fully robust to variation in marker density.

## Discussion

We described an approach to estimate the age of 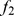 haplotypes, without making any prior assumptions about population structure or history. Though the age of any individual haplotype is uncertain, major features of the distribution of haplotype ages are detected, demonstrating qualitative differences between populations that are almost certainly due to past demographic events. The next important question is to what extent we can use these distributions as quantitative estimates of the ages of demographic events.

**Figure 5:**
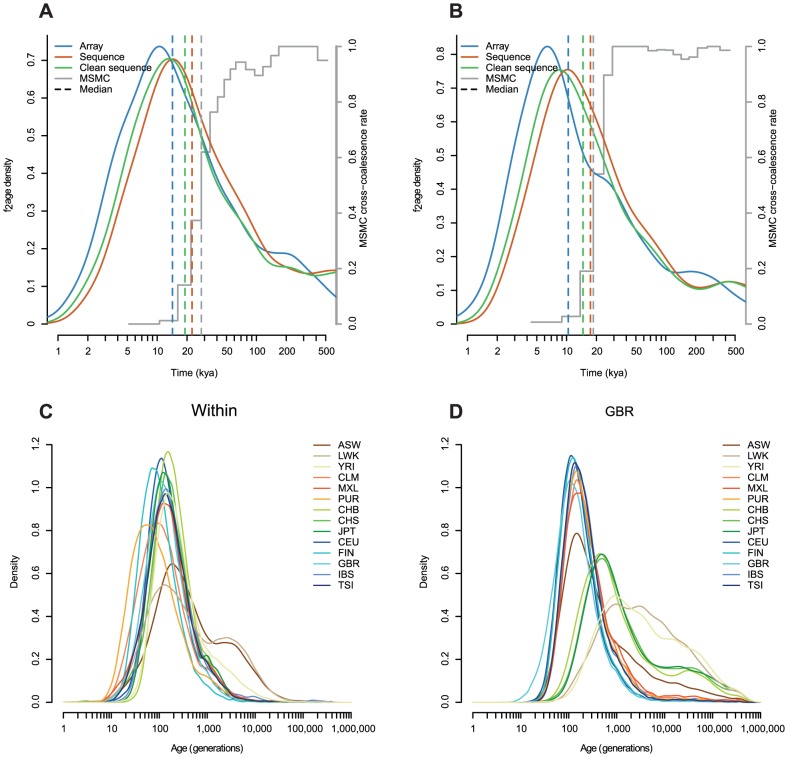
Comparison with MSMC, and the effect of estimating haplotypes with sequence data. **A**: The age distribution of 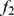 haplotypes shared between CHB and CEU estimated with array, sequence and “clean” sequence (with indels and low complexity regions removed; **Methods**). Coloured dashed lines show the medians of each distribution. The grey stepped line shows relative cross-population coalescence rates estimated by MSMC (S. Schiffels, personal communication), and the grey dashed line shows the earliest date in the oldest time interval where this rate is less that 0.5. In both cases, we assume 30 years per generation and 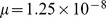. **B**: As in **A** but for 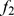 haplotypes shared between CHB and MXL, restricted to haplotypes where the MXL individual is inferred to be homozygous for Native American ancestry. **C–D**: Age distributions inferred using “clean” sequence data, comparable to Figure 3A 3A–B (Note the extended x-axis).

Consider the split between European and East Asian populations. Model based estimates of this split time have ranged from 14 to 40 thousand years ago (kya) [22]–[24]. However these are likely to be too low because they assumed a mutation rate, 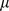, of 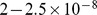 per-base per-generation, now thought to be an overestimate [19] and so a more reasonable range of estimates might be 22–80 kya. The nonparametric PSMC approach [25] estimated a split time of around 22 kya (if a lower mutation rate of 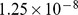 is used, 11 kya with the higher rate), and a similar method, MSMC, estimates a split time of 20–40 kya [26] (Figure 5A). Simulations suggest that, under a clean split model, the median of our estimated ages is close to or slightly below the split time, at least for recent splits (less than 1,000 generations; Figures S5 and S9). Comparing CEU to each of CHB, CHS and JPT, taking the median of our haplotype ages, and assuming a generation time of 30 years [27], would imply split times of 14, 17 and 18 kya respectively. Other European populations give different estimates, but mostly between 15 and 20 kya.

Similarly, when we looked at 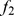 variants shared between East Asia and America (CHB-MXL, but restricting to regions homozygous for Native American ancestry in MXL; **Methods**), we found that the median age was around 10 kya, substantially more recent than the 20 kya split time estimated using MSMC [26] (Figure 5B). This seems low, given geological evidence that the Bering land bridge was submerged by 11–13 kya, although a seasonal or maritime route likely remained open after that time [28]–[30].

Our dates are all around or below the low end of published estimates, even after we take into account the fact that the median might be lower than the split time (we estimate about 11% lower for a 500-generation old split; Figure S5D). There are several non-exclusive explanations for this observation. First, post-split gene flow could explain the discrepancy. As we have greater power to detect 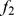 haplotypes if they are more recent, when the split is not clean many of the haplotypes we observe will derive from the post-split gene flow rather than from before the initial split (Figure S9B). In this scenario, we would be detecting the most recent haplotypes, and our dates would be closer to the most recent date of contact, rather than the initial split date.

An alternative explanation might be systematic errors in our estimates. As we described in the **Results**, the approach is sensitive to the estimated parameters 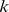, 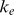 and 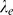. At the extreme, increasing 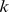 from 1.5 to its maximum value of 2 would increase the median age of CEU-CHB haplotypes from 14 kya to 17 kya. To investigate sensitivity to 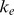 and 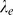, we repeated the analysis, but using sequence data rather than array data to find the length of the haplotypes (Figure 5). We note that when we estimated 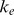 and 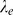 using sequence data they vary very little across chromosomes (Table S6). The ages estimated using sequence data were older than those estimated using array data (median age of CEU-CHB haplotypes 23 kya, Figure 5A–B). We might expect that sequence data, being denser than array data, would find haplotypes more accurately. However we would also expect that genotype errors, more common in sequence than array data, would make all haplotypes look older, by incorrectly breaking haplotypes. Removing indels and low complexity regions (LCRs; **Methods**) thought to be enriched for genotyping errors from the sequence data reduced the difference (median CEU-CHB age of 19 kya), suggesting that around half the increase in age is driven by errors. Further, the haplotype ages estimated from sequence data do not contain the very young (long) haplotypes within CLM, FIN and PUR, which we independently believe to be correct (Figure 5C), and also contain a long tail of extremely old haplotypes which seems unlikely (Figure 5D).

Another source of systematic errors could be the use of incorrect mutation or recombination rates. There is considerable uncertainty about the mutation rate in humans, but our approach is relatively insensitive to this, so if the true rate is higher than 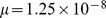 per-base per-generation then mutational clock based methods which scale linearly with mutation rate will overestimate the ages of events, thus reducing the discrepancy.

On the other hand, our approach might be sensitive to errors in the recombination map. We tested this by running simulations with a different genetic map to the HapMap map that we used to determine genetic length. We tested a population-based African American map [31], a map derived from an Icelandic pedigree [32] and a chimpanzee map from a small population [33], but none of these made a substantial difference to the results and we conclude that the length of the haplotypes we investigate is sufficiently large that they are robust to the uncertainty in the recombination map (Figure S10).

Finally, systematic errors might occur due to homoplasy (where the same mutation occurs independently on two different lineages). While this rate is expected to be low, it may be locally high in some parts of the genome, for example in CpG islands which have an order of magnitude higher mutation rate than the genomic background. If such false positives do occur, they would appear as very short haplotypes that we would infer to be very old, so they cannot explain our systematically lower ages. On the other hand, it is likely that some of the very old haplotypes we see are, in fact, due to repeat mutations and, in particular, this might explain some of the very old haplotypes discovered with sequence data.

However, while systematic biases in our estimates might explain some of the difference between our estimated ages and independent split time estimates, they are unlikely to explain the observation that the age distributions vary greatly between different pairs of populations. This strongly suggests that there is variation in the extent of gene flow. For example, Asian-FIN sharing seems to be more recent than other Asian-European sharing, suggesting relatively recent contact between East Asian and Finnish populations, compared to other European populations. It seems likely that worldwide demographic history is sufficiently complicated that trying to estimate a single Asian-European (or African-Non African) split time is futile, and that a complex model of many splits, migrations and admixtures is required to explain the relationship between different populations.

Ultimately, we would like to be able to make explicit estimates of parameters like historical effective population size, and the dates of demographic events. Though the approach we describe here is limited in in this respect, there is a clear path to extend it to do so. We could first use a similar approach to estimate the ages of variants at frequency three and higher. Then, treating the estimates of haplotype ages as estimates of coalescent times, we could use the empirical distribution of coalescent times to estimate population sizes and cross-population migration rates as a function of time. Another improvement would be to use information from the full likelihood surface for each haplotype, rather than just the point estimate of the age as we do here. Since, for large samples, we would have good estimates of recent coalescent rates, we expect that this approach would be very accurate at inferring recent history, making it a complementary approach to sequential Markovian coalescent based methods which are typically accurate in the ancient past, but less so for very recent history.

## Methods

### Definitions

Suppose we have a sample of size 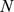 of genotypes from a single genetic region. Define an 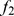 variant to be one which occurs exactly twice in the sample in different individuals. That is, either two individuals have genotype 1 and all the others have genotype 0, or two individuals have genotype 1 and the others have genotype 2. We assume that the minor allele is the derived allele. Under the neutral coalescent, for a sample of 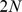 chromosomes, an 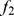 minor allele will be the derived allele with probability 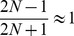 for large 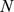 so this is a reasonable assumption for large samples.

Define an 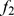 haplotype shared between chromosomes 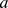 and 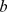 to be a region satisfying the following two conditions: 1) The time to the most recent common ancestor (TMRCA) of 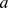 and 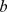 does not change over the region. 2) At one or more sites in the region, 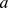 and 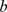 coalesce with each other before either of them coalesce with any other chromosome. In other words, they are unique genealogical nearest neighbours (Figure S1). We call the TMRCA of 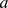 and 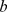 the age of the haplotype. Additionally, we say that individuals 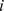 and 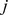 (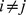) share an 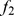 haplotype if 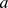 is one of 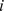
's two chromosomes and 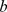 is one of 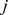
's two chromosomes.

The problem we solve is to find the 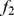 haplotypes and then estimate their ages. Since each 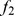 variant must lie in an 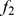 haplotype, the variants provide a simple way of detecting the haplotypes. We use the algorithm described in the main text to find regions which should be larger than the 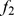 haplotypes. The next problem is to determine the likelihood of the age. We describe our approximate likelihood below but first, as an example, we describe exact inference in the absence of confounding factors.

### Exact case

Suppose we knew the exact genetic and physical lengths of an 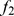 haplotype and the number of singletons it carries. Call these quantities 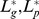 and 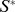. Let the age of this haplotype be 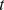 generations, or 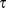 in coalescent time (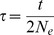). Then, for a randomly chosen 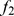 haplotype (but not a haplotype at a randomly chosen position, discussed in the next section), 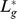 has an exponential distribution with parameter 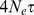 and 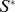 has a Poisson distribution with parameter 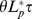 where 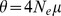 and 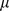 is the per-base per-generation mutation rate. Therefore (ignoring terms that do not depend on 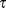), the log-likelihood of 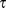 given 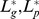 and 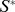 is
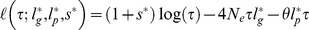
and the maximum likelihood estimator of 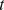 is therefore



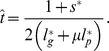



### Approximate likelihood for genetic length

There are two corrections to the likelihood for genetic length. The first relates to the ascertainment process of the haplotypes, and the second to the overestimate in the length due to the way we detect the endpoints.

The ascertainment problem is as follows. Suppose we pick a haplotype at random, then its length is exponentially distributed (i.e. gamma with shape parameter 1). However, if we pick a point on the sequence at random then the distribution of the length of the haplotype in which it falls is gamma distributed with shape parameter 2. This is an example of the “inspection paradox” and it is because in the second case, we are sampling haplotypes effectively weighted by their length. In our case, we detect haplotypes if they contain one or more 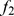 variants. Therefore the probability that we find a haplotype is increasing with its physical length (because longer haplotypes are more likely to carry 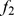 variants), but sub-linearly. The probability also increases with genetic length, but in a complex way that depends on the variation of recombination and mutation rate along the genome, the age of the haplotype and the demographic history of the population. For example, in a constant sized population, older haplotypes are likely to have longer branches above them, and therefore to have more 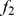 variants, but in an expanding population the opposite may be true. Rather than trying to take all of these effects into account, we made the simplifying assumption that we could model the genetic length 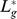 as a gamma distribution with shape parameter 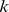 where 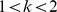 and rate 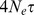. Simulations suggested that 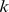 around 1.5 was optimal (Figure S11), and we used this value throughout.

The second correction involves the overestimate of genetic length. We tried to detect the ends of the haplotype by looking for inconsistent homozygote genotypes, but of course in practice, after the end of the 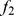 haplotype, there will be some distance before reaching such a site. This (genetic) distance 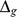 is the amount by which we overestimate the length of the haplotype. We estimate the distribution of 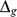 for a given sample by sampling pairs of genotype vectors, then sampling sites at random and computing the sum of genetic distance to the first inconsistent homozygote site on either side. We then fit a gamma distribution with (shape, rate) parameters 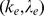 to this distribution, for each chromosome. The likelihood of 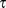 is given by the convolution density of 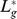 and 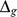,
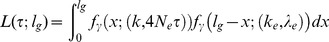
(1)where 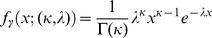 is the density of a gamma distribution with (shape, rate) parameters 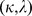. This integral, and therefore the loglikelihood 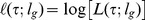 can be expressed in terms of the confluent hypergeometric function 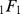 (ignoring terms that do not depend on 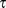),



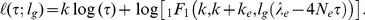
(2)Where, recall, we assume 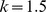. Note that if we replace 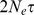 with 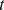, and drop constant terms, then we get an expression for the likelihood of 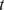 that does not depend on 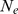, so our estimate of time in generations does not depend on 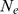.
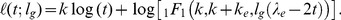
(3)


Finally, note that the rate at which recombination events occur on the branch connecting the two shared haplotypes is 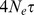. We assume that the first such event marks the end of the haplotype. However, there is a non-zero probability that a recombination event occurring on this branch does not change the MRCA of 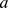 and 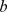. Simulations suggest that for large numbers of chromosomes, this probability is extremely small (Figure S12) and so we assume it is 0. In practice, for small samples, this might be a non-negligible effect.

### Approximate likelihood for singleton count

Recall that the physical length of the shared haplotype is 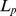 bases. We assume that we can find this exactly. Then assuming a constant mutation rate 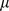 per base per generation, the sum of the number of singletons on the shared haplotypes, 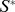 has a Poisson distribution with parameter 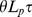, where 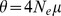.

Now consider the distribution of singletons on the unshared haplotypes. To approximate this distribution, we make the following three assumptions: 1) There is no recombination on the unshared haplotypes over the region. 2) No other lineage coalesces with the shared haplotype before it is broken. 3) The distribution of the time to first coalescence of the unshared haplotypes is exponential with parameter 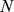 (Recall that 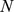 is the number of sampled individuals). In fact the true distribution is a mixture of exponentials but the approximation at least matches the correct mean, 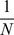 [34]. The variance is too small because of the first assumption, however.

Consider one of the unshared haplotypes. Conditional on the time (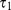) at which it first coalesces with any other haplotype, the number of singleton mutations it carries is Poisson with parameter 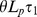 and so, using the assumptions above, the unconditional distribution is geometric (on 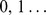) with parameter 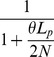. Therefore the distribution of the number of mutations on both unshared haplotypes, 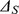, is the sum of two geometric distributions which is negative binomial with parameters 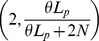. The density of the total number of singletons, 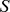 is the convolution of these two densities
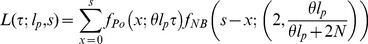
(4)where 
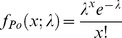
 is the density of a Poisson distribution with parameter 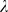 and 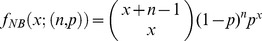 is the density of a negative binomial distribution with parameters 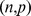. As with the genetic length, we can write this in terms of 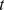, the haplotype age in generations,



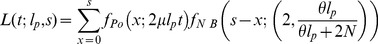
(5)In practice we assume 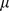 is known and estimate 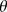 separately for each individual, for each chromosome, by counting the number of singletons, multiplying by the number of chromosomes in the sample, and dividing by the chromosome length. Then for each pair, we use use the average of these values in Equation 5. A more accurate approach would be to compute the likelihood as a double convolution over the distribution of both haplotypes with different values for 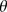. An extension would be to estimate 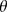 separately for different regions of the genome.

### Approximate full likelihood

We can now write the approximate log-likelihood for 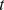 as the sum of Equation 3 and the log of Equation 5, assuming that the recombination process is independent of the mutational process,
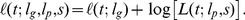
(6)


We maximise it numerically with respect to 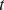 in order to find the maximum likelihood estimate (MLE). It is possible for this likelihood to be bimodal, in which case we might find a local but not global optimum. However, this seems to be rare.

### 1000 Genomes data

The 1000 Genomes data was obtained from ftp://ftp.1000genomes.ebi.ac.uk/vol1/ftp/. The phase 1 release sequence data is in phase1/analysis_results/integrated_call_sets, and the array data is in phase1/analysis_results/supporting/omni_haplotypes. In order to generate the “clean” sequence data, we removed any sites that fell in the list of low complexity regions found in technical/working/20140224_low_complexity_regions/hs37d5-LCRs.txt. Functional annotations are in phase1/analysis_results/functional_annotation. Detailed explanations of the annotations can be found there, but briefly the classifications are as follows:

- Loss-of-function: Includes premature stop codons, and essential splice site disruptions.
- Coding: Variants in coding regions.
- Functional noncoding: Including variants in noncoding RNAs, promoters, enhancers and transcription factor binding sites.
- Unannotated: Any variant not included in any of the above categories.

We included haplotypes in more than one of these categories if they contained multiple variants.

### Code

All the code we used to run simulations and analyse the 1000 Genomes data is available from www.github.com/mathii/f2.

## Supporting Information

Figure S1Example of an 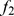 haplotype. Recombination events are shown as a series of marginal trees, as we move left to right along a sequence, so the tree above point 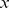 is constant between 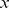 and 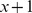. In the blue region, 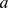 and 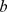 share an 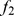 haplotype. At sites 2 and 3, 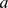 and 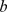 are unique nearest neighbours. A mutation at site 2 (green), will be detected as an 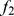 variant. At site 4, they are no longer unique nearest neighbours, but the TMRCA is unchanged. At site 5, the TMRCA has changed and the haplotype breaks. In the other direction, at site 1 the haplotype breaks because the TMRCA of 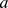 and 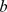 changes, even though they are still unique nearest neighbours. We count this as breaking the haplotype, even though we cannot detect this event.(PDF)

Figure S2Confidence intervals. **A**: Coverage of approximate confidence intervals. We performed simulations as described in Figure 2, but only for chromosome 20. We computed approximate 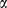-confidence intervals using the 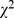 approximation to the distribution of the log-likelihood. This figure shows the proportion of true haplotype ages that lie inside their approximate confidence intervals. **B**: Confidence intervals for the simulations in Figure 2. For each haplotype, we plot its true age against the upper and lower end of the two-tailed 95% confidence interval.(PDF)

Figure S3The contributions of different terms of the likelihood. This shows plots comparable to Figure 2, based on whole-genome simulations of 100 individuals. Grey dots show the estimated age of each 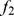 haplotype against the true age. The blue line is a qq plot of the distribution of the MLEs (from 1% to 99% quantiles) Each subfigure shows the result of using different information. **A**, **C** and **E** use just the genetic length and **B**, **D** and **F** use both the genetic length and the number of singletons. **A** and **B** show the results if the true values are used in the likelihood. **C** and **D** show the corresponding results when the observed length is used without accounting for the overestimate, **E** and **F** (the same as Figure 2A) show the full likelihood, including the correction to 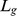.(PDF)

Figure S4The effect of demography on the accuracy of inference. These plots were generated with the same parameters as Figure 2 (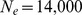, 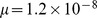), but show only chromosome 20. Grey dots show the estimated age of each detected 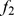 haplotype against the true age (in generations). The blue line is a qq plot of the distribution of the MLEs (from 1% to 99% quantiles). We simulated different demographic scenarios. **A**: A bottleneck which reduces the population by 99% between 14 and 17 generations in the past (dotted lines). **B**: A population of size 14,000 which split into two isolated populations, each of size 14,000, 560 generations ago (dotted line). **C**: A population growing exponentially by about 0.02% per generation, to a present size of 140,000. **D**: A population where 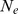 is actually 140,000 but we ran inference assuming it to be 14,000.(PDF)

Figure S5The effect of demography on inferred age distributions. Solid lines show the density estimate of the distribution of the MLEs of the ages of detected 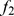 haplotypes under different demographic scenarios. Dashed lines show the true distributions. Parameters as in Figure 2. **A**: A recent bottleneck. Dotted lines show the time of the bottleneck (population size reduced by 99% for 3 generations). **B**: Population split 1120 generations ago with no subsequent migration. The dotted line shows the time of the population split. The blue line shows the estimated age of 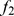 variants shared within a population and the red line the estimated age of 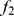 variants shared between populations. The vertical red dotted line shows the median of this distribution and the black dotted line shows the split time. **C**: Age distributions for different bottlenecks. Different colours show results for bottlenecks at different times. As in **A**, dashed lines show the true distribution of 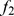 ages and solid lines of the same colour show the estimated distributions. Parameters as in **A**, but now the bottleneck reduces the population size by 90% for 5 generations and the recombination map is from chromosome 10 instead of 20. **D**: Median within and between population ages, both estimated (solid line) and true (dashed line), for different split times. As in **B**, but again using the chromosome 10 recombination map.(PDF)

Figure S6

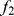
 variant sharing across 1000 Genomes individuals. Colours from white to blue to red show the number of 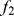 variants shared between each pair of individuals, normalised by the total in each row. Individuals are ordered by populations, but only by sample name within each population.(PNG)

Figure S71000 Genome Project 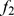 haplotype MLE age distributions. For the nine populations not included in Figure 3. Each of these subfigures shows the distribution of ages of haplotypes shared between one population and each of the others.(PDF)

Figure S8GBR-American sharing. The density of the ages of 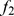 haplotypes shared between GBR (UK) and each of CEU (NW European), CLM (Columbian), IBS (Spanish), MXL (Mexican), PUR (Puerto Rican) and TSI (Tuscan). See Table 1 for more complete descriptions of the populations.(PDF)

Figure S9The effect of post-split migration. Comparison of the distribution of the age of 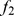 variants shared between populations (blue) and the gene flow estimated by MSMC with 4 haplotypes (red). 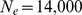, 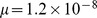, using the chromosome 20 recombination map. In each case, the blue dashed line shows the median of the 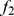 age distribution. **A**: A scenario where the two populations split 1120 generations ago (black dashed line). **B**: A scenario where two populations split 1120 generations ago, but there is migration at a rate of 1% per year for 560 generations (black dashed lines at 560 and 1120 generations). Note that in **B**, the peak of the 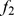 age density is shifted to the left relative to **A**, indicating that many of the 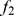 variants shared between populations derive from post-split migration rather than predating the split.(PDF)

Figure S10The effect of errors in the recombination map. We simulated haplotypes with a different recombination map to the one used to determine genetic length. The left column shows true versus estimated ages for detected haplotypes, and a qq plot of the MLEs, as in Figure 2A. The right column shows the coverage of the asymptotic confidence intervals as in Figure S2A. Each row shows the results of simulations using a different map (references in main text), but in every case the HapMap combined map was used to determine the genetic length of the detected haplotypes: **A,B**: Simulated using the HapMap map. **C,D**: Simulated using a map derived from African Americans. **E,F**: Simulated using a map derived from an Icelandic pedigree. **G,H**: Simulated using a map derived from chimpanzees, rescaled to have the same total length as the human map. In each case, we simulated Chromosome 20 for 100 individuals with 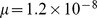 per-base per-generation and 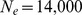.(PDF)

Figure S11Effect of varying 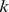. This figure shows the effect on the density estimate of varying 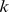, the shape parameter of the gamma distribution used to model the genetic length of the haplotypes. This shows qq plots generated from simulations as in Figure 2, but for chromosome 20 only, for 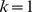, 1.5 and 2.(PDF)

Figure S12Probability that recombinations do not change the TMRCA. We used simulations to estimate the probability that the first recombination on the branch between two haplotypes which are nearest neighbours does not change their TMRCA. For varying sample sizes 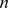, we simulated sequences with recombination using the SMC' algorithm [35]. We find pairs of nearest neighbours, then count along the sequence until the first recombination on the branch connecting them. The plot shows the probability that this recombination does not change the TMRCA (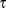) of those two samples, as a function of 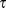. Solid circles show 5% and 95% quantiles of the distribution of coalescence times. The dashed lines show the theoretical lower bounds on this probability; 
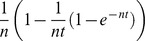
, exact for 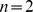, which is achieved if there are no coalescences on the tree except the one between the two nearest neighbour lineages. The numbers in brackets in the legend show the probability that a recombination does not change the TMRCA, averaged over all events. Note that as 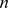 increases, for fixed 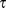, the probability of not changing the TMRCA decreases, but in addition the distribution of 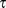 becomes smaller which also decreases the overall probability of not changing the TMRCA.(PDF)

Table S11000 Genomes 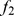 haplotype total counts. Counts of 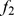 haplotypes shared between each pair of populations.(TXT)

Table S21000 Genomes 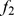 haplotype mean counts. Mean number of 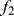 haplotypes shared between each pair of individuals, for each pair of populations.(TXT)

Table S31000 Genomes 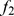 haplotype medians. Median estimated age (in generations) of the MLE of the 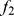 haplotypes shared between each pair of populations, using array data to estimate the haplotypes.(TXT)

Table S41000 Genomes 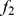 haplotype 5% quantiles. 5% quantile of estimated age (in generations) of the MLE of the 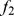 haplotypes shared between each pair of populations, using array data to estimate the haplotypes.(TXT)

Table S51000 Genomes 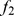 haplotype 95% quantiles. 95% quantile of estimated age (in generations) of the MLE of the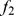 haplotypes shared between each pair of populations, using array data to estimate the haplotypes.(TXT)

Table S6Estimated error parameters for 1000 Genomes data. We show error parameters 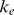 and 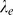 for each chromosome estimated with both array and sequence data, and 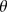 (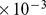) estimated by counting singletons, for each chromosome, showing the median value for non-African (N.Afr) and African (Afr) populations separately. Note that there is substantial variation in error parameters between chromosomes using array but not sequence data, suggesting that this is due to variations in the density of markers on the array, when we use array data to estimate the haplotypes.(TXT)
