# Supplementary figures and images for: Demography and the Age of Rare Variants
Source: PLoS Genet. 2014 Aug 7;10(8):e1004528. doi: 10.1371/journal.pgen.1004528 (PMC4125085; doi:10.1371/journal.pgen.1004528)

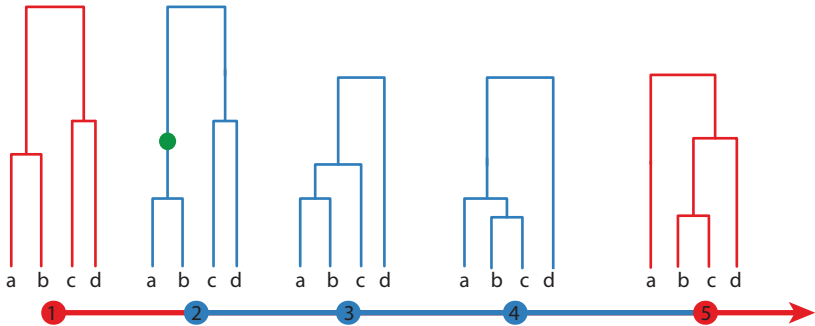

Supplement: Figure S1 — Example of an haplotype. Recombination events are shown as a series of marginal trees, as we move left to right along a sequence, so the tree above point is constant between and . In the blue region, and share an haplotype. At sites 2 and 3, and are unique nearest neighbours. A mutation at site 2 (green), will be detected as an variant. At site 4, they are no longer unique nearest neighbours, but the TMRCA is unchanged. At site 5, the TMRCA has changed and the haplotype breaks. In the other direction, at site 1 the haplotype breaks because the TMRCA of and changes, even though they are still unique nearest neighbours. We count this as breaking the haplotype, even though we cannot detect this event. (PDF) [file pgen.1004528.s001.pdf]

**A**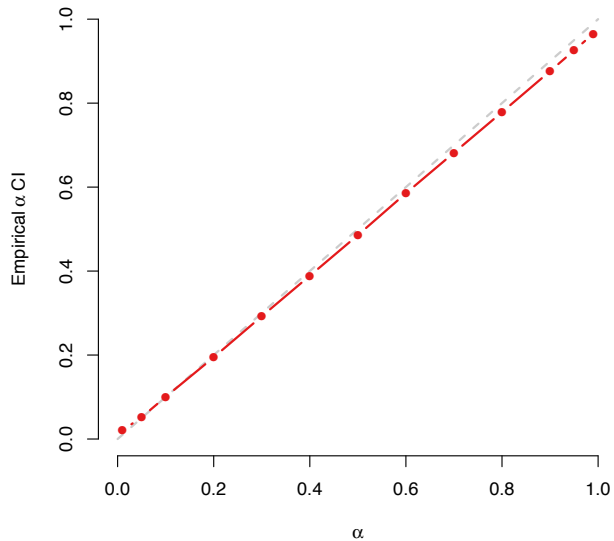**B**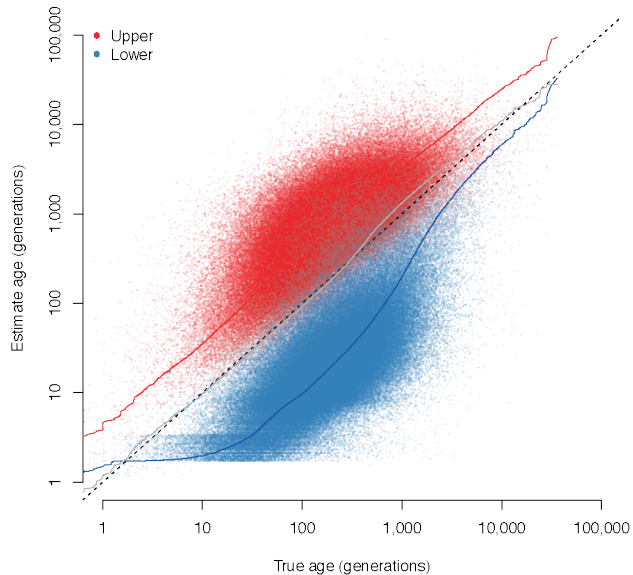

Supplement: Figure S2 — Confidence intervals. A: Coverage of approximate confidence intervals. We performed simulations as described in Figure 2, but only for chromosome 20. We computed approximate -confidence intervals using the approximation to the distribution of the log-likelihood. This figure shows the proportion of true haplotype ages that lie inside their approximate confidence intervals. B: Confidence intervals for the simulations in Figure 2. For each haplotype, we plot its true age against the upper and lower end of the two-tailed 95% confidence interval. (PDF) [file pgen.1004528.s002.pdf]

**A**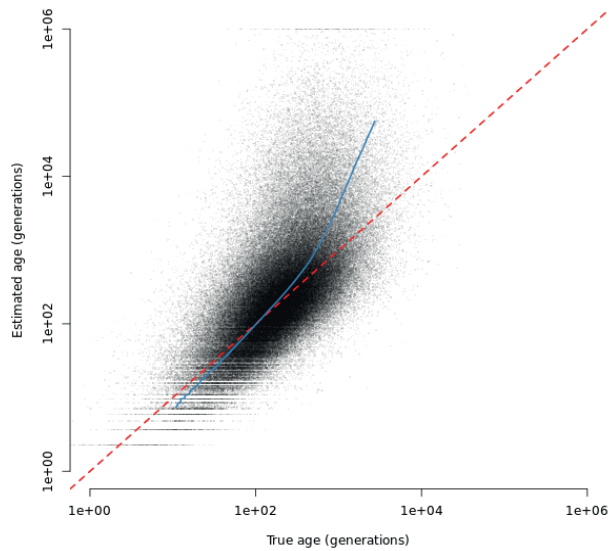**B**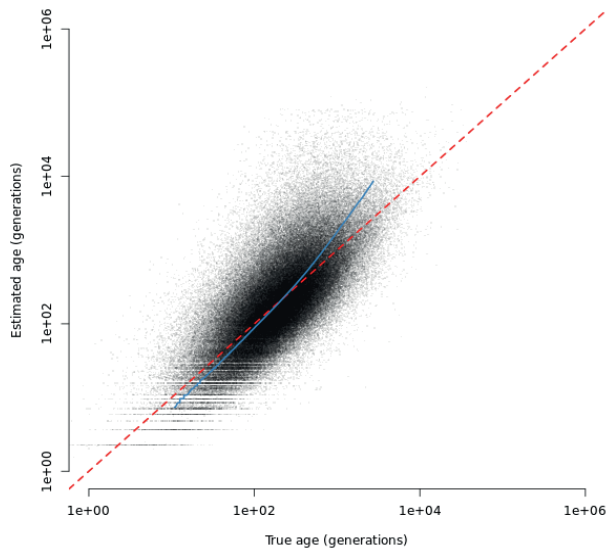**C**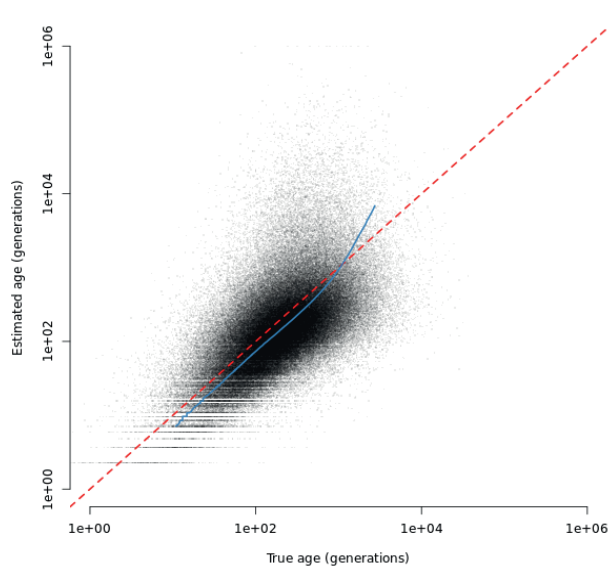**D**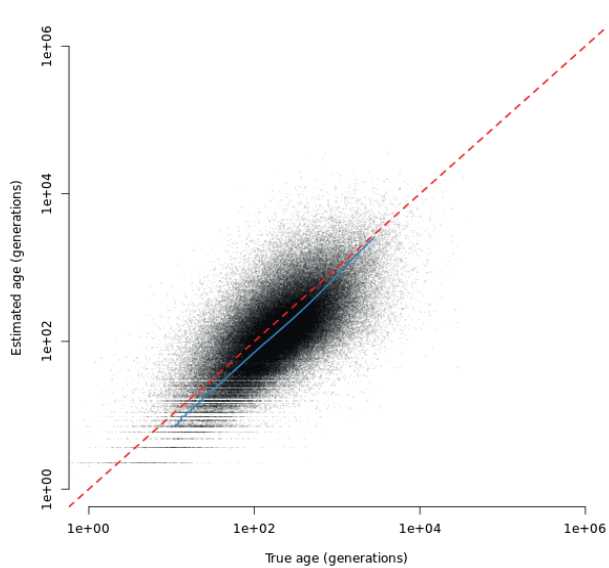**E**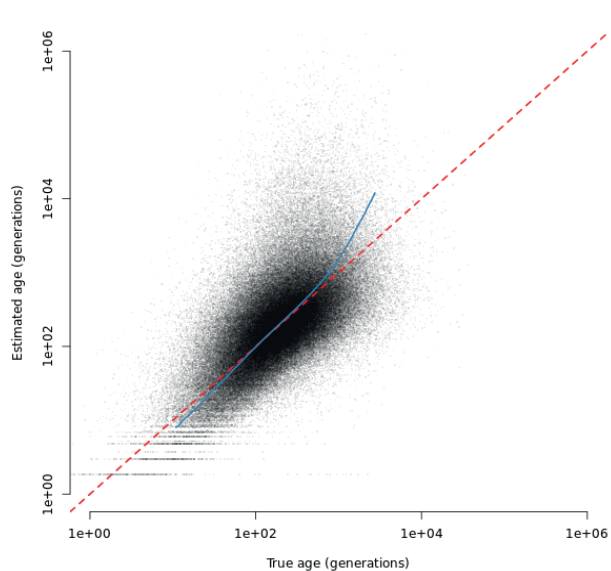**F**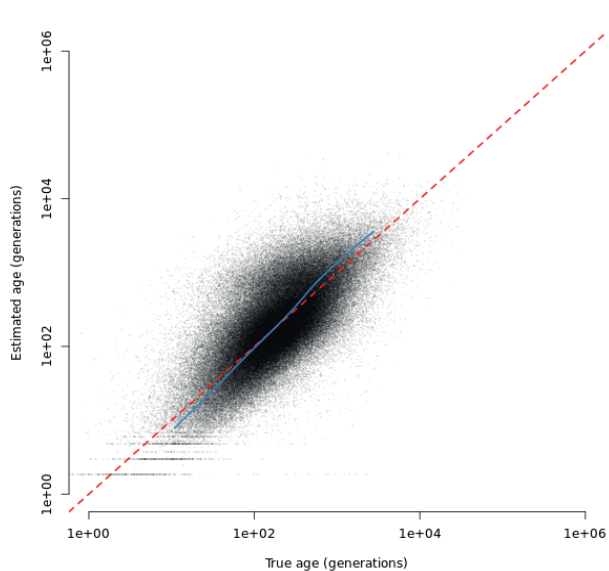

Supplement: Figure S3 — The contributions of different terms of the likelihood. This shows plots comparable to Figure 2, based on whole-genome simulations of 100 individuals. Grey dots show the estimated age of each haplotype against the true age. The blue line is a qq plot of the distribution of the MLEs (from 1% to 99% quantiles) Each subfigure shows the result of using different information. A, C and E use just the genetic length and B, D and F use both the genetic length and the number of singletons. A and B show the results if the true values are used in the likelihood. C and D show the corresponding results when the observed length is used without accounting for the overestimate, E and F (the same as Figure 2A) show the full likelihood, including the correction to . (PDF) [file pgen.1004528.s003.pdf]

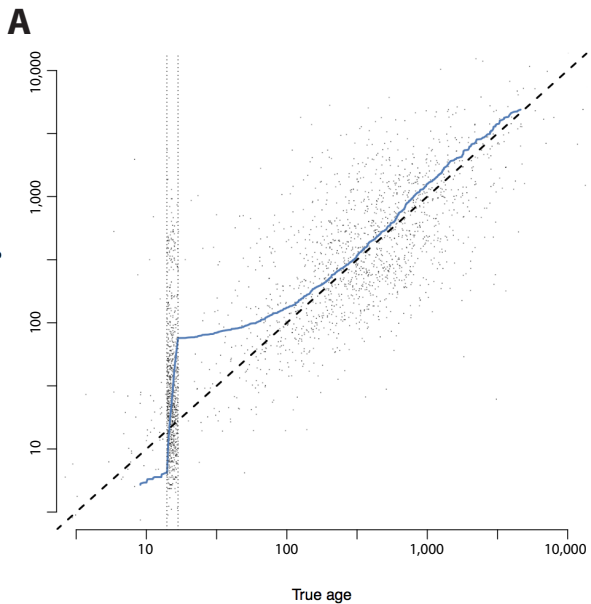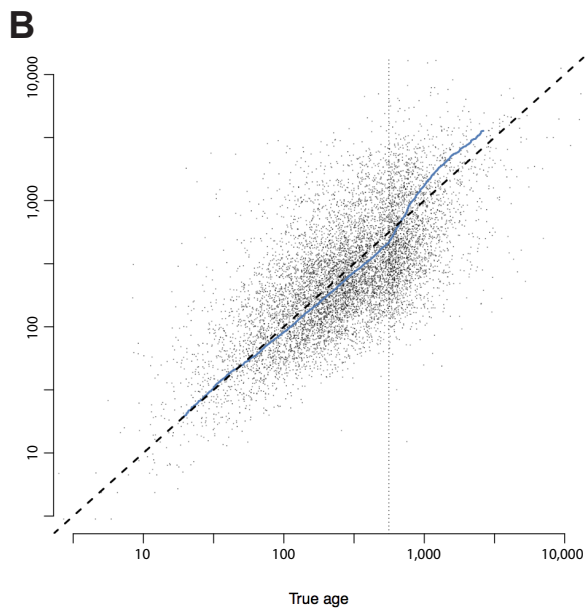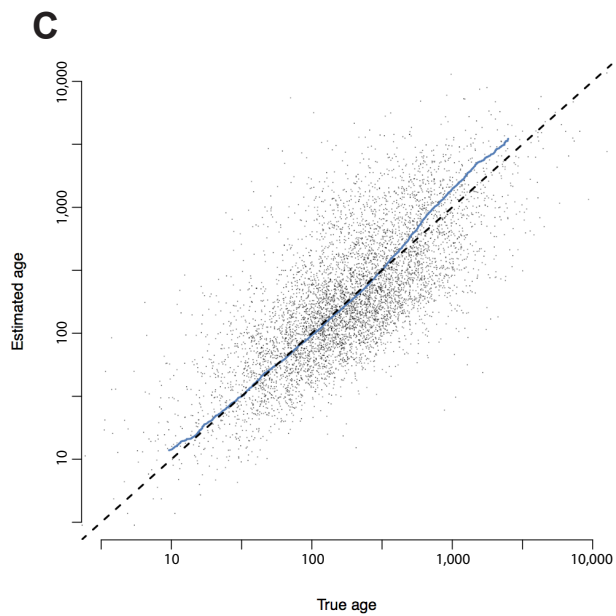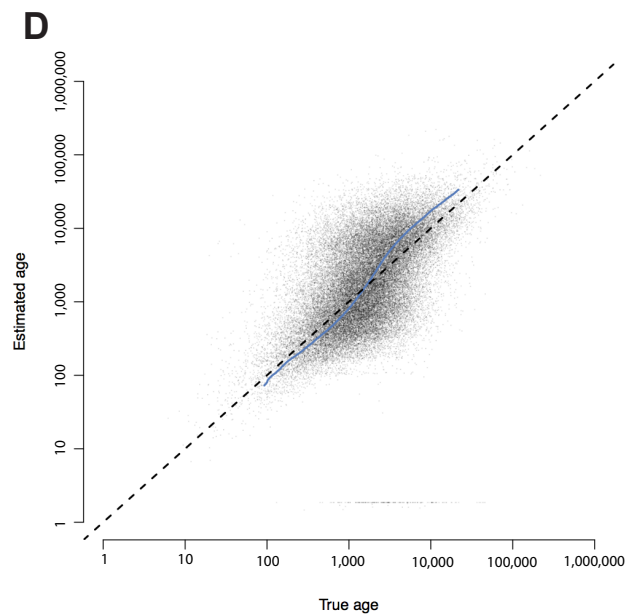

Supplement: Figure S4 — The effect of demography on the accuracy of inference. These plots were generated with the same parameters as Figure 2 (, ), but show only chromosome 20. Grey dots show the estimated age of each detected haplotype against the true age (in generations). The blue line is a qq plot of the distribution of the MLEs (from 1% to 99% quantiles). We simulated different demographic scenarios. A: A bottleneck which reduces the population by 99% between 14 and 17 generations in the past (dotted lines). B: A population of size 14,000 which split into two isolated populations, each of size 14,000, 560 generations ago (dotted line). C: A population growing exponentially by about 0.02% per generation, to a present size of 140,000. D: A population where is actually 140,000 but we ran inference assuming it to be 14,000. (PDF) [file pgen.1004528.s004.pdf]

**A**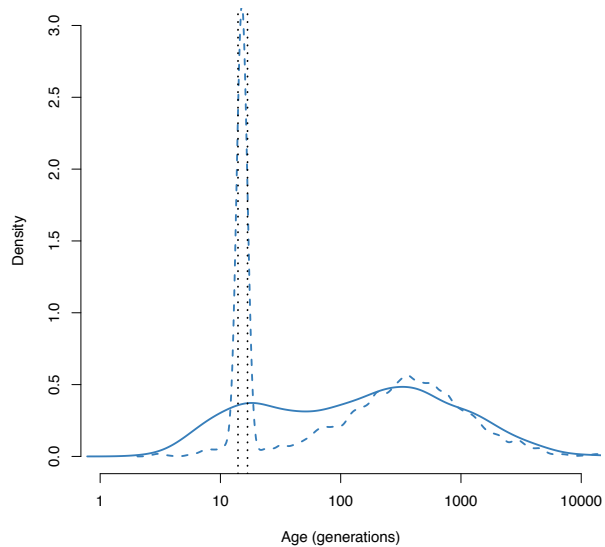**B**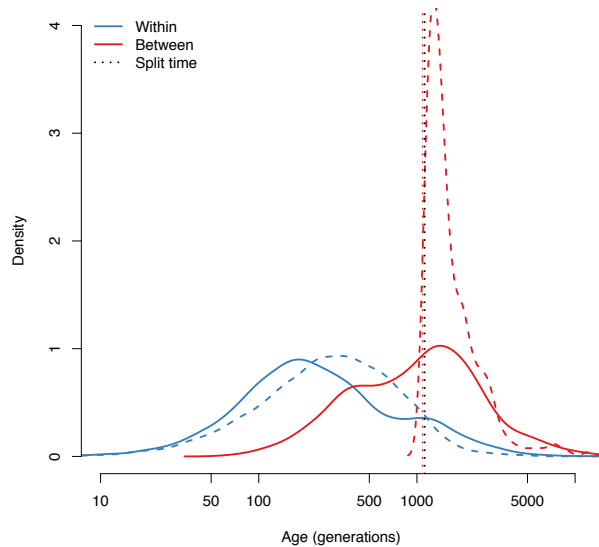**C**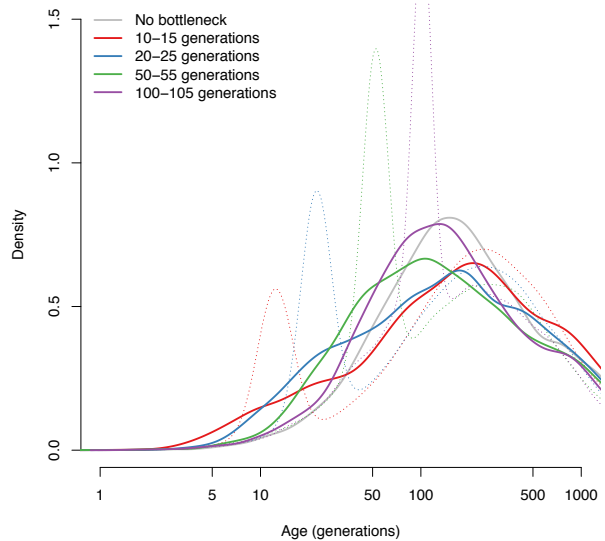**D**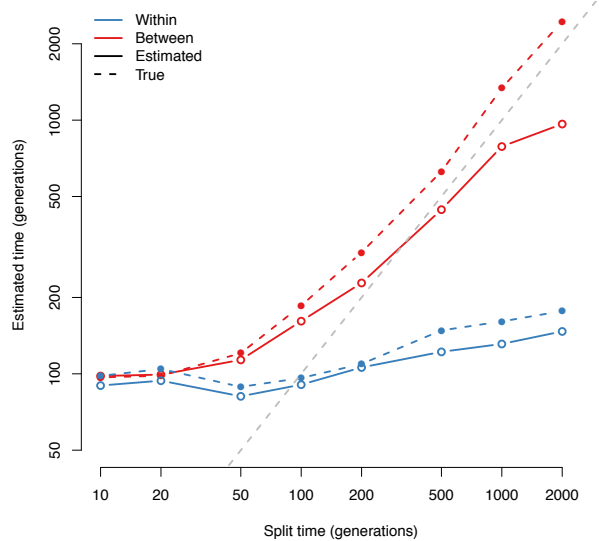

Supplement: Figure S5 — The effect of demography on inferred age distributions. Solid lines show the density estimate of the distribution of the MLEs of the ages of detected haplotypes under different demographic scenarios. Dashed lines show the true distributions. Parameters as in Figure 2. A: A recent bottleneck. Dotted lines show the time of the bottleneck (population size reduced by 99% for 3 generations). B: Population split 1120 generations ago with no subsequent migration. The dotted line shows the time of the population split. The blue line shows the estimated age of variants shared within a population and the red line the estimated age of variants shared between populations. The vertical red dotted line shows the median of this distribution and the black dotted line shows the split time. C: Age distributions for different bottlenecks. Different colours show results for bottlenecks at different times. As in A, dashed lines show the true distribution of ages and solid lines of the same colour show the estimated distributions. Parameters as in A, but now the bottleneck reduces the population size by 90% for 5 generations and the recombination map is from chromosome 10 instead of 20. D: Median within and between population ages, both estimated (solid line) and true (dashed line), for different split times. As in B, but again using the chromosome 10 recombination map. (PDF) [file pgen.1004528.s005.pdf]

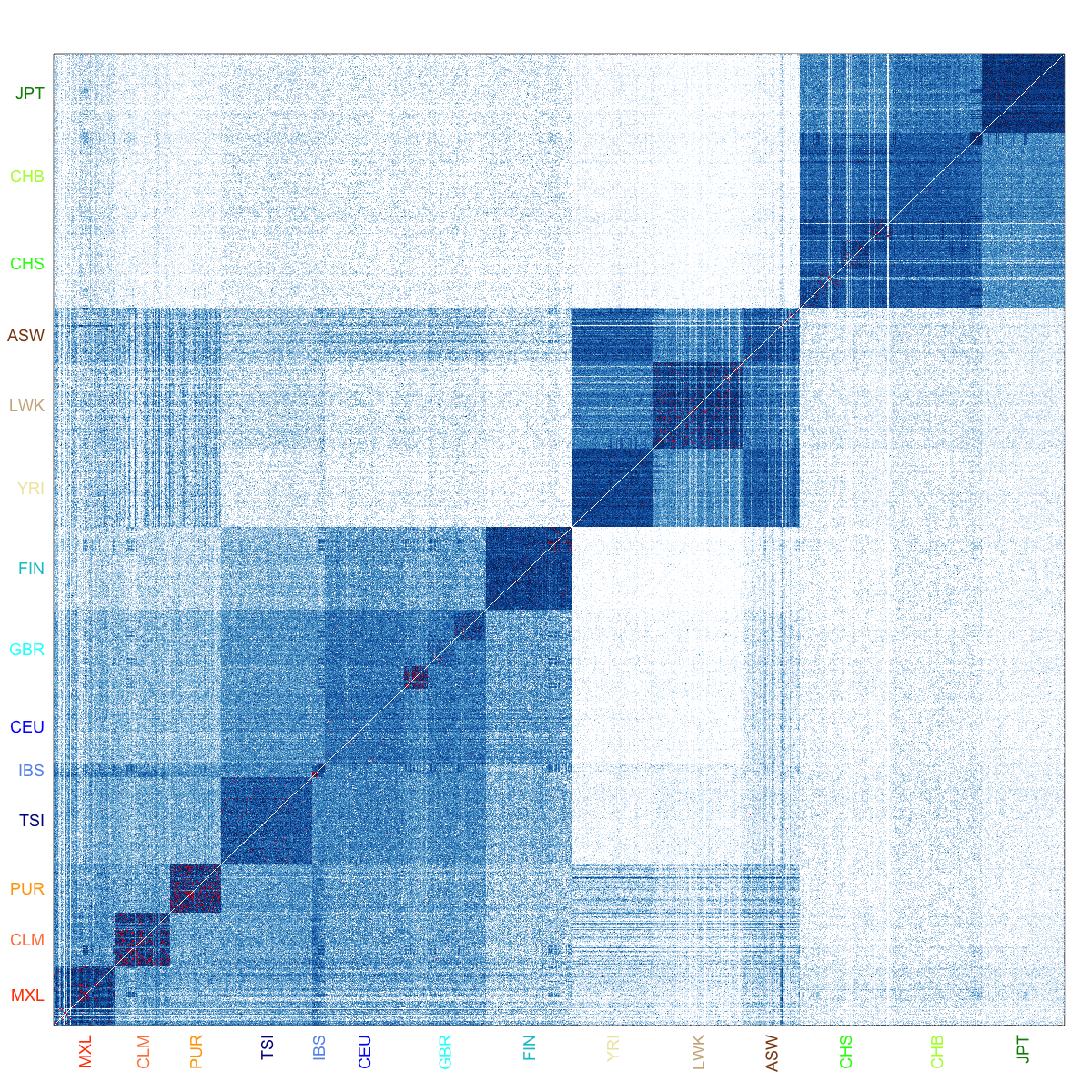

Supplement: Figure S6 — variant sharing across 1000 Genomes individuals. Colours from white to blue to red show the number of variants shared between each pair of individuals, normalised by the total in each row. Individuals are ordered by populations, but only by sample name within each population. (PNG) [file pgen.1004528.s006.png]

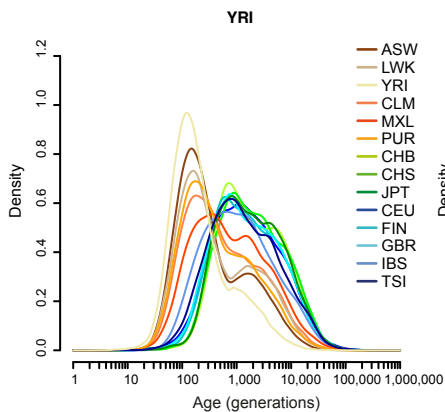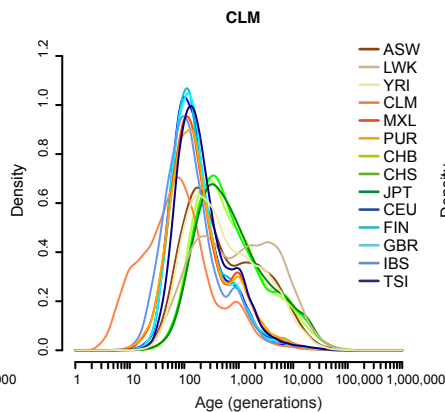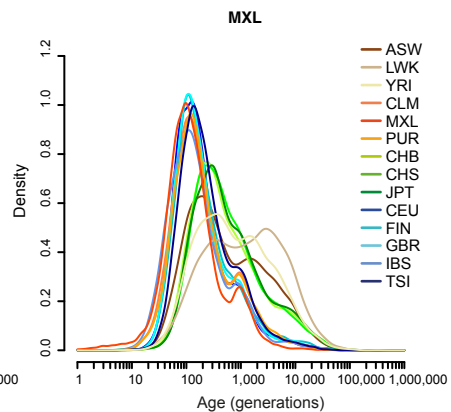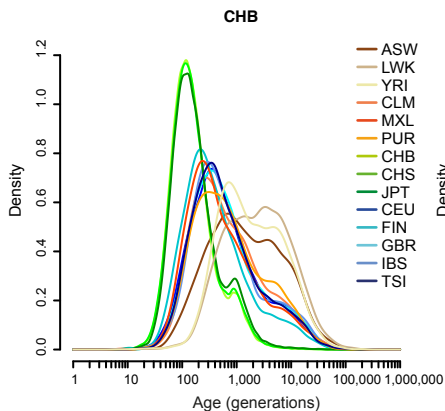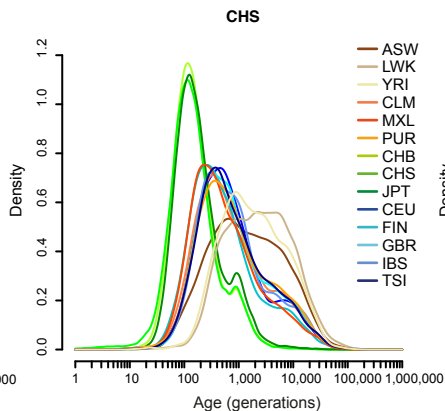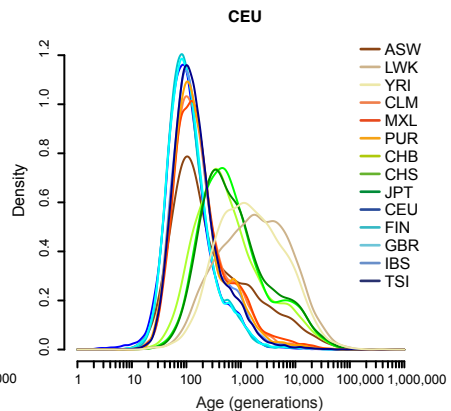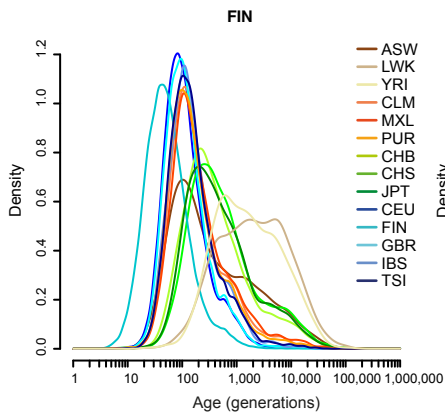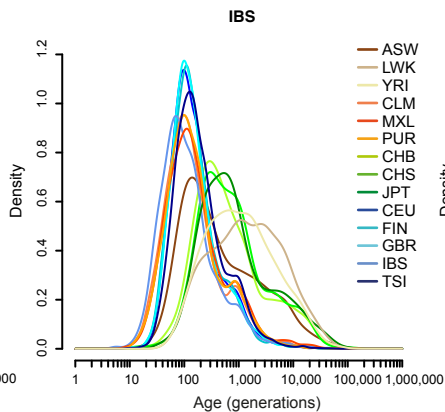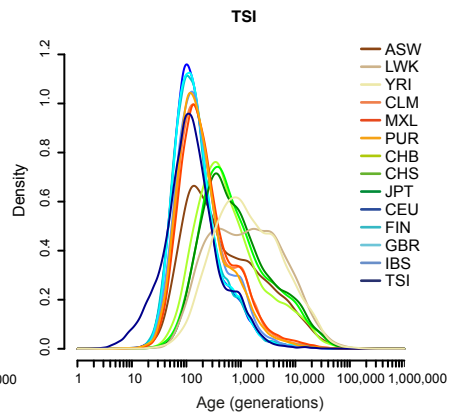

Supplement: Figure S7 — 1000 Genome Project haplotype MLE age distributions. For the nine populations not included in Figure 3. Each of these subfigures shows the distribution of ages of haplotypes shared between one population and each of the others. (PDF) [file pgen.1004528.s007.pdf]

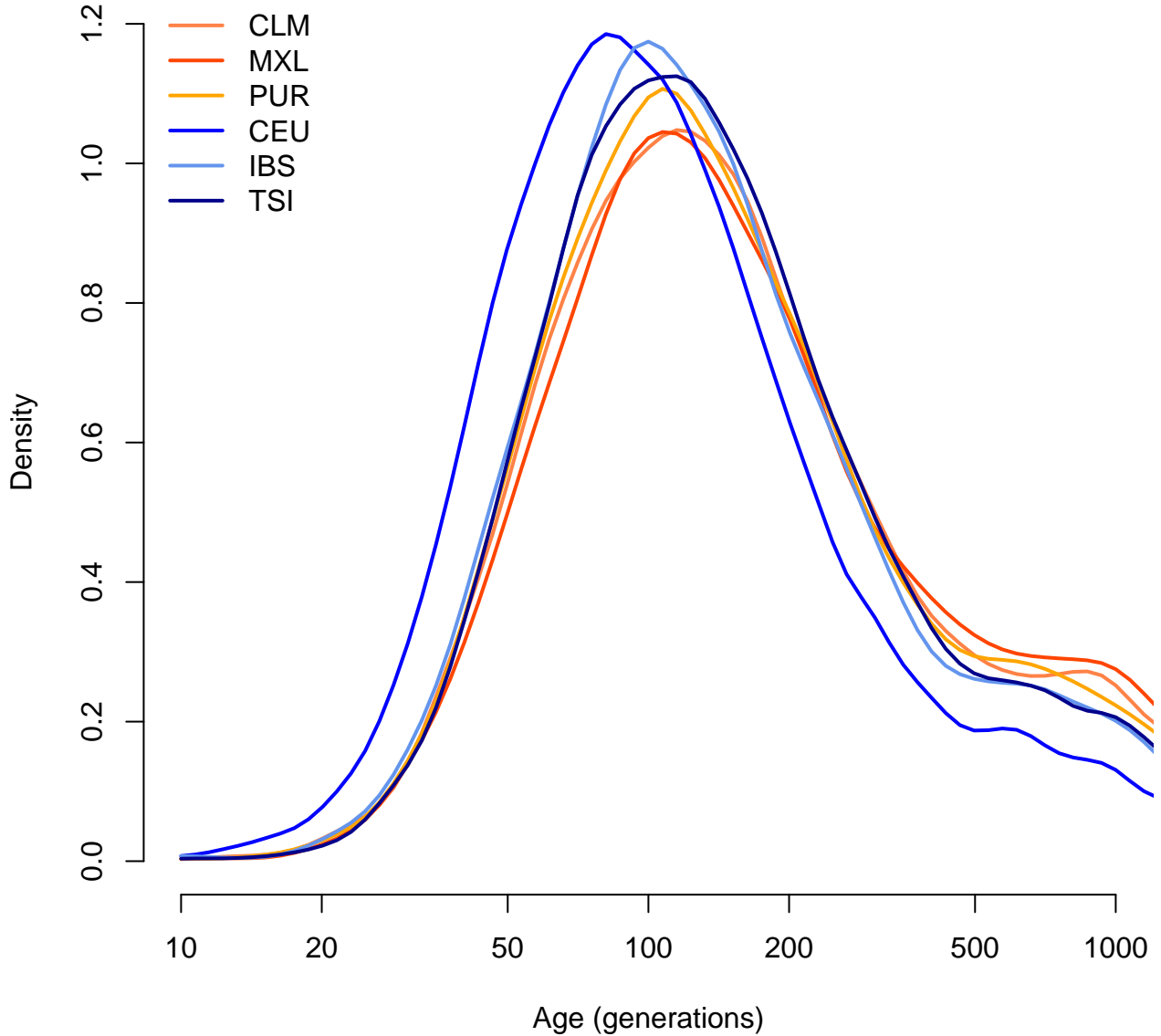

Supplement: Figure S8 — GBR-American sharing. The density of the ages of haplotypes shared between GBR (UK) and each of CEU (NW European), CLM (Columbian), IBS (Spanish), MXL (Mexican), PUR (Puerto Rican) and TSI (Tuscan). See Table 1 for more complete descriptions of the populations. (PDF) [file pgen.1004528.s008.pdf]

**A**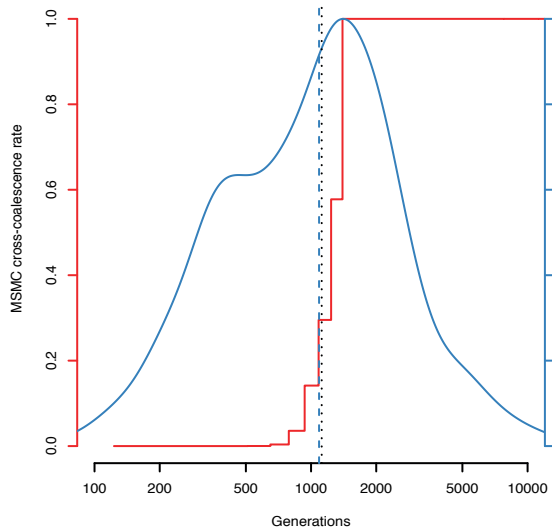**B**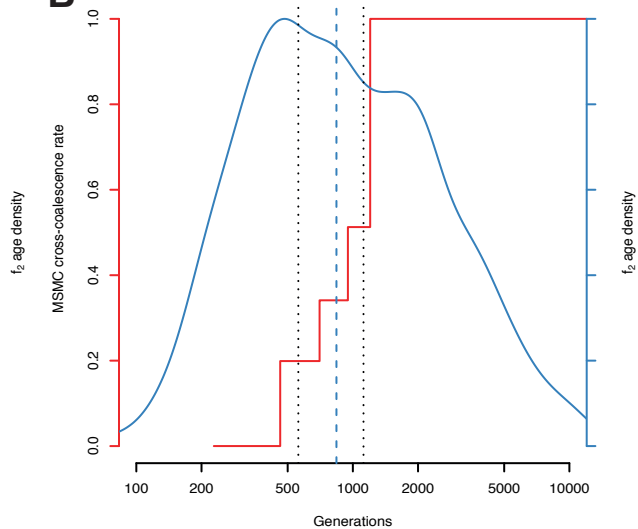

Supplement: Figure S9 — The effect of post-split migration. Comparison of the distribution of the age of variants shared between populations (blue) and the gene flow estimated by MSMC with 4 haplotypes (red). , , using the chromosome 20 recombination map. In each case, the blue dashed line shows the median of the age distribution. A: A scenario where the two populations split 1120 generations ago (black dashed line). B: A scenario where two populations split 1120 generations ago, but there is migration at a rate of 1% per year for 560 generations (black dashed lines at 560 and 1120 generations). Note that in B, the peak of the age density is shifted to the left relative to A, indicating that many of the variants shared between populations derive from post-split migration rather than predating the split. (PDF) [file pgen.1004528.s009.pdf]

HapMap Map

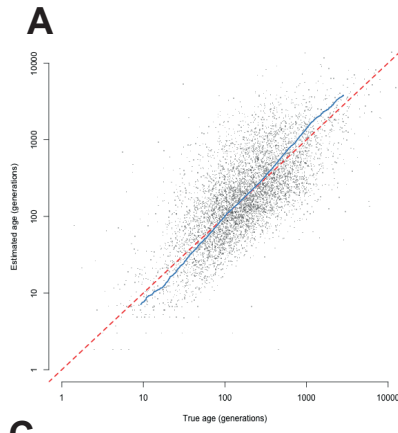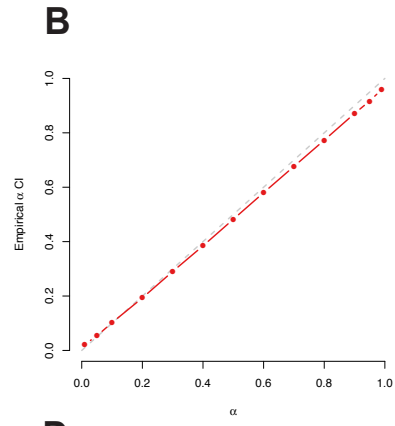

African-American Map

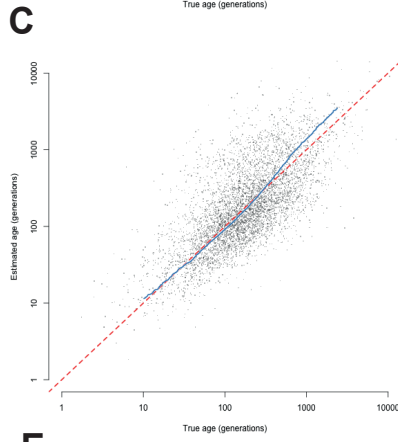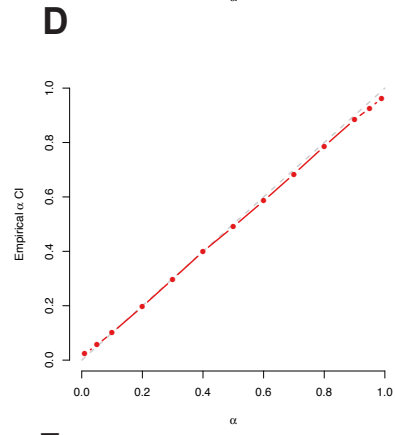

Decode Map

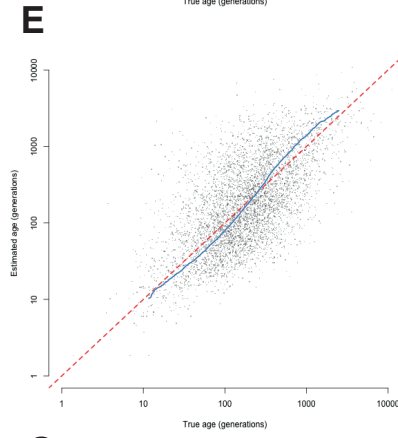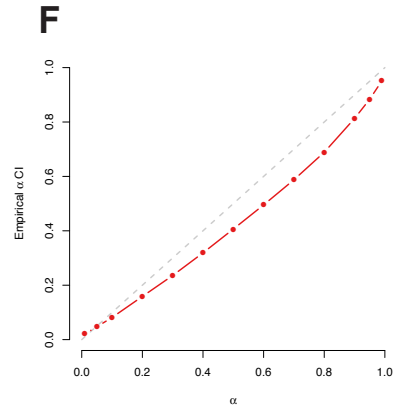

Chimpanzee Map

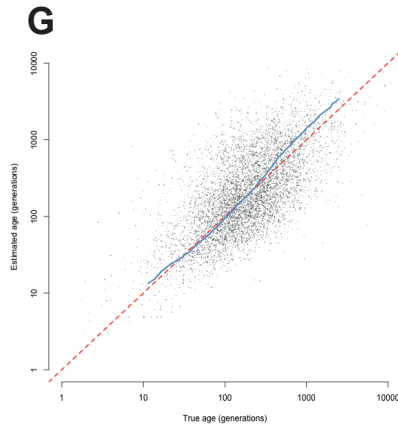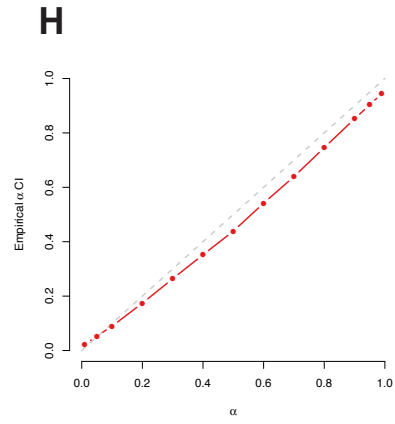

Supplement: Figure S10 — The effect of errors in the recombination map. We simulated haplotypes with a different recombination map to the one used to determine genetic length. The left column shows true versus estimated ages for detected haplotypes, and a qq plot of the MLEs, as in Figure 2A. The right column shows the coverage of the asymptotic confidence intervals as in Figure S2A. Each row shows the results of simulations using a different map (references in main text), but in every case the HapMap combined map was used to determine the genetic length of the detected haplotypes: A,B: Simulated using the HapMap map. C,D: Simulated using a map derived from African Americans. E,F: Simulated using a map derived from an Icelandic pedigree. G,H: Simulated using a map derived from chimpanzees, rescaled to have the same total length as the human map. In each case, we simulated Chromosome 20 for 100 individuals with per-base per-generation and . (PDF) [file pgen.1004528.s010.pdf]

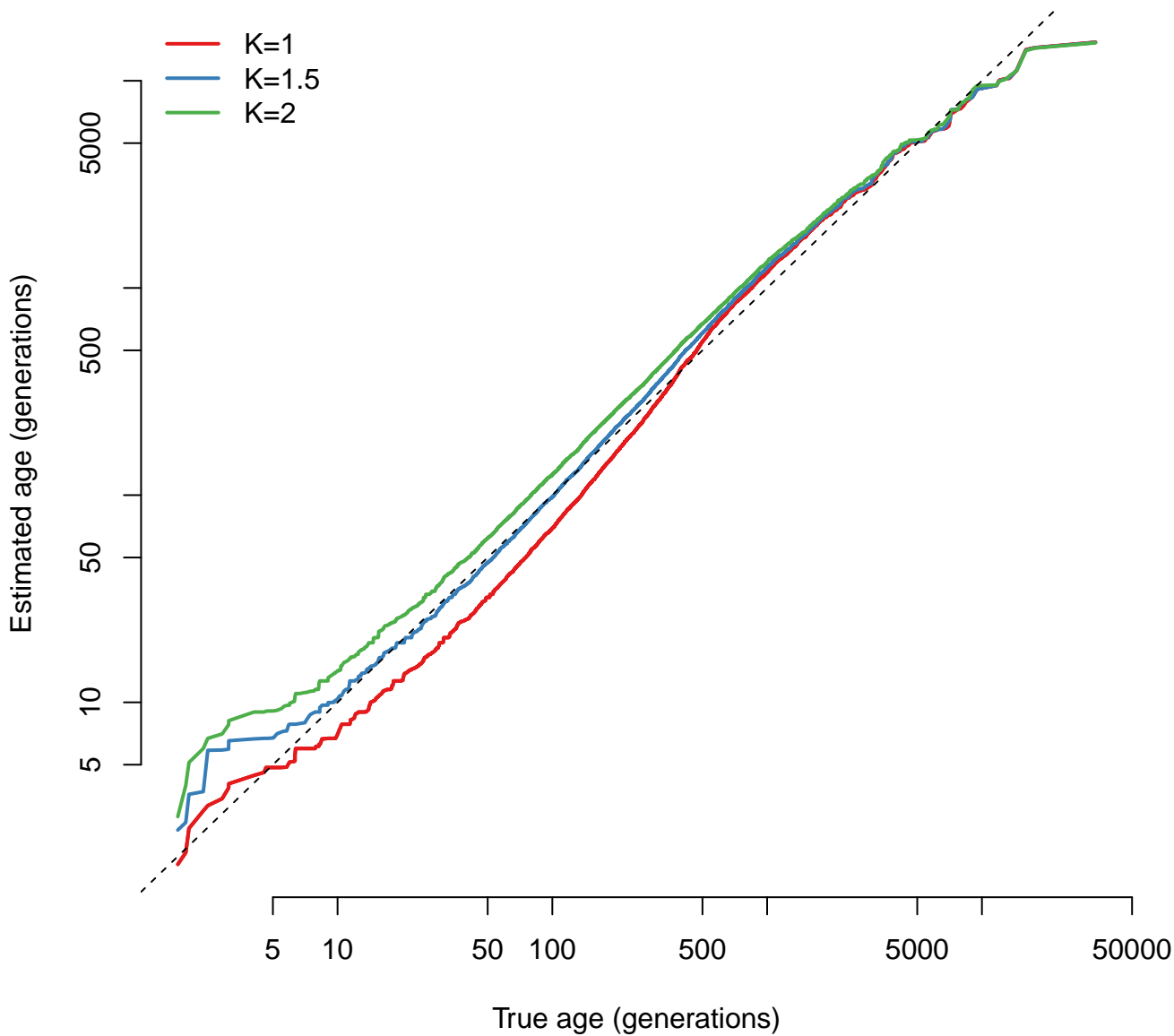

Supplement: Figure S11 — Effect of varying . This figure shows the effect on the density estimate of varying , the shape parameter of the gamma distribution used to model the genetic length of the haplotypes. This shows qq plots generated from simulations as in Figure 2, but for chromosome 20 only, for , 1.5 and 2. (PDF) [file pgen.1004528.s011.pdf]

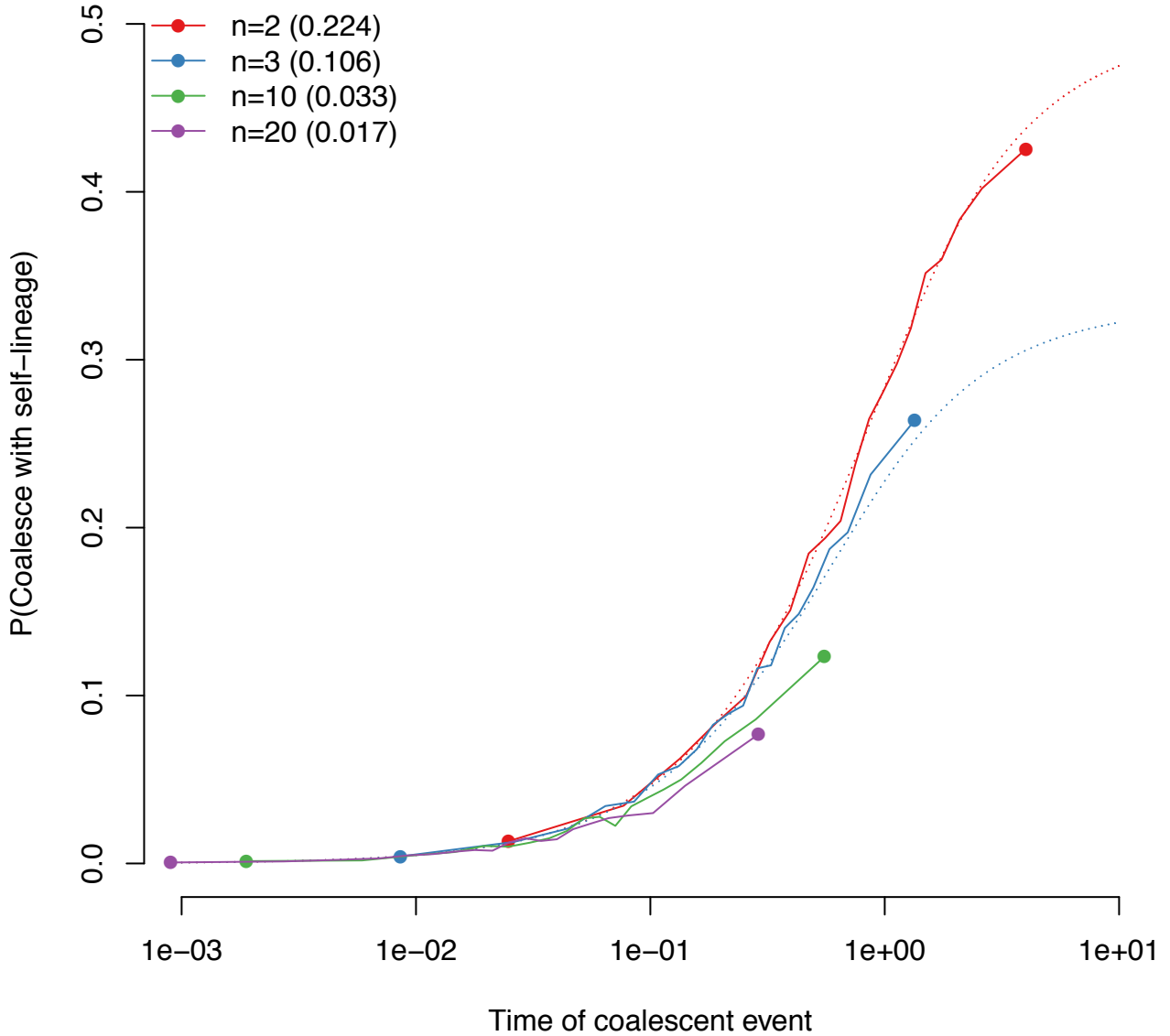

Supplement: Figure S12 — Probability that recombinations do not change the TMRCA. We used simulations to estimate the probability that the first recombination on the branch between two haplotypes which are nearest neighbours does not change their TMRCA. For varying sample sizes , we simulated sequences with recombination using the SMC' algorithm [35]. We find pairs of nearest neighbours, then count along the sequence until the first recombination on the branch connecting them. The plot shows the probability that this recombination does not change the TMRCA () of those two samples, as a function of . Solid circles show 5% and 95% quantiles of the distribution of coalescence times. The dashed lines show the theoretical lower bounds on this probability; , exact for , which is achieved if there are no coalescences on the tree except the one between the two nearest neighbour lineages. The numbers in brackets in the legend show the probability that a recombination does not change the TMRCA, averaged over all events. Note that as increases, for fixed , the probability of not changing the TMRCA decreases, but in addition the distribution of becomes smaller which also decreases the overall probability of not changing the TMRCA. (PDF) [file pgen.1004528.s012.pdf]
